# Phylodynamics of Hepatitis C Virus Subtype 2c in the Province of Córdoba, Argentina

**DOI:** 10.1371/journal.pone.0019471

**Published:** 2011-05-18

**Authors:** Viviana E. Ré, Andrés C. A. Culasso, Silvia Mengarelli, Adrián A. Farías, Fabián Fay, María B. Pisano, Osvaldo Elbarcha, Marta S. Contigiani, Rodolfo H. Campos

**Affiliations:** 1 Facultad de Ciencias Médicas, Instituto de Virología, Universidad de Córdoba, Córdoba, Argentina; 2 Cátedra de Virología, Facultad de Ciencias Químicas, Universidad Católica de Córdoba, Córdoba, Argentina; 3 Cátedra de Virología, Facultad de Farmacia y Bioquímica, Universidad de Buenos Aires, Buenos Aires, Argentina; 4 Servicio de Gastroenterología, Hospital San Roque, Córdoba, Argentina; 5 Laboratorio CIBIC, Centro de Diagnóstico Moleculares, Rosario, Argentina; Saint Louis University, United States of America

## Abstract

The Hepatitis C Virus Genotype 2 subtype 2c (HCV-2c) is detected as a low prevalence subtype in many countries, except in Southern Europe and Western Africa. The current epidemiology of HCV in Argentina, a low-prevalence country, shows the expected low prevalence for this subtype. However, this subtype is the most prevalent in the central province of Córdoba. Cruz del Eje (CdE), a small rural city of this province, shows a prevalence for HCV infections of 5%, being 90% of the samples classified as HCV-2c. In other locations of Córdoba Province (OLC) with lower prevalence for HCV, HCV-2c was recorded in about 50% of the samples. The phylogenetic analysis of samples from Córdoba Province consistently conformed a monophyletic group with HCV-2c sequences from all the countries where HCV-2c has been sequenced. The phylogeographic analysis showed an overall association between geographical traits and phylogeny, being these associations significant (α = 0.05) for Italy, France, Argentina (places other than Córdoba), Martinique, CdE and OLC. The coalescence analysis for samples from CdE, OLC and France yielded a Time for the Most Common Recent Ancestor of about 140 years, whereas its demographic reconstruction showed a “lag” phase in the viral population until 1880 and then an exponential growth until 1940. These results were also obtained when each geographical area was analyzed separately, suggesting that HCV-2c came into Córdoba province during the migration process, mainly from Europe, which is compatible with the history of Argentina of the early 20th century. This also suggests that the spread of HCV-2c occurred in Europe and South America almost simultaneously, possibly as a result of the advances in medicine technology of the first half of the 20th century.

## Introduction

Hepatitis C Virus (HCV) is an enveloped single-stranded positive RNA virus [1] that currently infects 3% of the world's population [2]. In about 80% of infections, it causes a silent chronic hepatic illness that can lead to fibrosis, cirrhosis and hepatocellular carcinoma [3]. It is taxonomically classified into six genotypes (1 to 6) and many subtypes (a, b, c, etc.) based on the phylogenetic analysis of partial genomic sequences (E1/core and NS5B region) [4]. Genotypes 3 and 6 are thought to have a South Eastern Asian origin, whereas genotypes 1, 2 and 4 may have an African origin, being genotype 2 more prevalent in Western Africa [5]. The current cosmopolitan distribution of certain subtypes such as 1a, 1b, 2a, 2b, 2c and 3a is the result of blood transfusions, intravenous drug abuse and invasive medical and surgical procedures that have been common for the last 80 years [4]. However, the mode of transmission in the ancestral endemic regions is still unknown [5]. The role of human migration in the early and recent spread of some HCV subtypes, such as HCV-2c, has been discussed in recent studies on European populations [6]. Moreover, most of the sequences for HCV-2c deposited in Genbank, including the D50409 “BEBE-1” HCV-2c reference sequence [7], belong to Europe, mainly France and Italy [8], [9]. However, HCV-2c has been spotted all around the world as a low-prevalence HCV subtype (http://hcv.lanl.gov).

The occurrence of HCV-2c in Argentina has a particular hallmark, since its prevalence has an interesting geographical correlation: a North-to-South gradient of HCV-2c prevalence has been identified in the Province of Córdoba. A genotype survey carried out with a restriction fragment length polymorphism (RFLP) assay showed that 90% of HCV-infected patients (5% of the population) in Cruz del Eje City (North region of Córdoba Province) have HCV-2a/c [10]. In Córdoba City (which is located in the Central region and is the Capital of the Province and the second most populated city of Argentina) and its surrounding locations, 50% of the patients carrying HCV are infected with HCV-2a/c [11]. Since the RFLP assay is carried out in a highly conserved region of the HCV genome, Ré *et al.* [12] used additional sequence information (Core region) in a sub-sample of these patients and found that they were infected with HCV-2c.

### Aim

In this study we aimed to elucidate the origin and dissemination of HCV-2c in the Province of Córdoba, Argentina, by applying phylodynamic and phylogeographic methods.

## Materials and Methods

### Ethic Statement

All patients included in this study signed a written informed consent according to local regulations. This work is part of a research project inscribed and approved by the ethics committee of the Health Ministry of the Province of Córdoba.

### Samples

Samples were obtained from two sources: 49 samples came from an epidemiological survey carried out in 2004 on a representative sample of 1,471 out of 28,166 inhabitants of Cruz del Eje city [10] and 26 samples came from patients from the general population attending the Virology Service of the “Instituto Dr. J. M. Vanella” from 1999 to 2008, who at the time of the study were living in Córdoba City and other small cities and towns of the province.

### RT-PCR and Sequencing

Serum RNA was extracted with TRIzol Reagent (Invitrogen) following the manufacturer's indications. Reverse transcription was carried out with MMLV-RT (Promega) with Random Hexamer Primers using the manufacturer's protocol. Polymerase chain reaction (PCR) for a 367-bp product of the NS5B region was carried out as described elsewhere [13] using Green Go Taq Reagents (Promega). Nested PCR for a 677-bp fragment in the E1/E2 region was carried using the primers depicted in Table 1. All PCR products were purified and direct-sequenced by an ABI automatic sequencer.

**Table 1:** HCV Subtype 2c E2 Primers.

Name	Sequence	Start*	End*	Round
2c-ES	5′-TGG GAT ATG ATG ATG AAC TGG T-3′	1298	1319	First
2c-EA	5′-AAT GCC ACA GCC TGT ARG GRT A-3′	2204	2183	First
2c-IS	5′-GGG GGC GTG GGC MAA RGT-3′	1435	1452	Second
2c-IA	5′-TGA ARC AGT CYG TGG GGC A-3′	2111	2093	Second

*Positions relative to BEBE1 Subtype 2c HCV reference genome (accession # D50409).

### Statistical Analyses

Mean age of patients from both Cruz del Eje city and other locations of Córdoba was estimated with the patients' age in 2004. The difference between means was tested with a non-paired Student's *t*-test after testing normality of distribution and equality in variances with Graph Pad Prism 4.02 for Windows.

### Sequences

For subsequent analyses, both E2 and NS5B sequences were prepared in several data sets.

Sequences obtained in this study were classified into two data sets according to their geographical origin: **CdE** (Cruz del Eje city) and **OLC** (Other Locations of Córdoba): several towns and cities of the Province of Córdoba).

Sequences downloaded from Genbank were used to build other two data sets: the **Genotype Reference Dataset**, which contained the sequences that represent the panel of Reference HCV genomes in the NCBI Viral Genotyping Tool plus two more partial NS5B sequences belonging to subtype 2i (marked with *): Subtype 1a: AF009606, AF011751, AF271632; Subtype 1b: D90208, AB049088, AF356827, AF139594; Subtype 1c: D14853, AY051292; Subtype 2a: D00944, AF169004, AB047639, AB047641; Subtype 2b: D10988, AF238486, AB030907, AY232730; Subtype 2c: D50409; Subtype 2i: DQ155561, D86530*, L48499*; Subtype 2k: AB031663; Subtype 3a: D17763, D28917, AF046866; Subtype 3b: D49374; Subtype 3k: D63821; Subtype 4a: Y11604; Subtype 5a: Y13184, AF064490; Subtype 6a: Y12083; Subtype 6b: D84262; Subtype 6d: D84263; Subtype 6g: D63822; Subtype 6h: D84265; Subtype 6k: D84264; and the **HCV-2c Dataset**, which contained sequences that overlapped the NS5B region studied in this work and that were already classified as subtype 2c when they were deposited in Genbank or reported in relevant publications [14], [15]. This last data set contained sequences from several countries: Lithuania (1), Russia (2), Canada (3), Estonia (3), Martinique (3), Argentina (from places other than Córdoba Province) (5), Germany (6), Italy (11) and France (24). The French sequences were included in another data set, named **Fr**, used in a coalescent re-analysis for comparative purposes.

All Genbank sequences tagged as region E1–E2 for HCV subtype 2c were discarded since they overlapped the region analyzed only in the amino terminal segment of E2 (hypervariable region 1).

### Phylogenetic Analyses

The phylogeny of the sequences was constructed using the Maximum Likelihood, Neighbor Joining and Parsimony methods.

The Maximum Likelihood tree was constructed with the PhyML 3.0 software [16] using nonparametric bootstrapping for branch support (100 pseudo replica). The model of nucleotide substitution was selected according to the Akaike Information Criterion implemented in ModelTest 3.7 software [17] for each data set analyzed.

Neighbor Joining trees were constructed with MEGA 4 Software [18] setting Maximum Composite Likelihood (Tamura & Nei 1993 substitution model with co-estimation of rate parameters) as the model of nucleotide substitution with heterogeneous rate among sites using the gamma distribution for the relative rate with the alpha shape parameter set according to ModelTest 3.7 estimation. Branch support was assessed by bootstrap analysis over 1000 pseudo replica.

Most parsimonious trees found with TNT software [19] using a New Technology Search (combining Sectorial Search, Tree Drifting and Tree Fusing) were used to construct a strict consensus tree.

### Coalescent Analyses

Dating of Most Recent Common Ancestor (t_MRCA_) of sequences was carried out for NS5B data sets by Monte Carlo Markov Chains (MCMC) Bayesian coalescent analysis implemented in BEAST version 1.6.0 software [20]. All BEAST chains were run for 5×10^7^ generations in order to achieve an Effective Sample Size (ESS)>200. The model of nucleotide substitution was set according to ModelTest results (see “Phylogenetic Analyses”). Since the time interval covered by the sequences of HCV-2c for the NS5B region with known sampling date is about 10 years, the substitution rate parameter could not be reliably estimated, and thus an external substitution rate was used. A substitution rate of 5×10^−4^ (Standard Deviation = 9×10^−5^) substitutions per site per year (s/s/y) was set according to a previous estimation [21] for the NS5B region. Bayesian Skyline Plots (BSPs) were run under the two molecular clock models: Strict and Relaxed Uncorrelated Lognormal. The resulting BSPs were compared by Bayes Factors [22] and the best clock model was selected for the estimation of t_MRCA_.

The E2 region substitution rate was also estimated by MCMC analysis. In this case, the t_MRCA_ calculated for the NS5B region was set as prior for the BSP analyses of the E2 data sets. These BSPs were run under the two above-mentioned molecular clock models. Once again, the best clock model was selected by Bayes Factor. Finally, the substitution rate was estimated from the selected BSPs.

All BEAST run logs were analyzed with TRACER program version 1.5 (Available from http://beast.bio.ed.ac.uk/Tracer) after discarding 2% of the run length as burn-in.

### Phylogeographic Analysis

The association between phylogeny and geography was statistically assessed in a Bayesian framework implemented in the BaTS program [23], which takes into account the phylogenetic uncertainty in the analysis. A new data set containing all the sequences of the CdE, OLC and HCV-2c data sets was analyzed with BEAST using BSP as demographic model. Eleven states were defined, named after the origin of the sample: Lithuania, Russia, Canada, Estonia, Martinique, Argentina (places other than Córdoba province), Germany, Italy, France, Cruz del Eje and Other Locations of Córdoba. Two BSPs were run under the different clock models (Strict Clock and Relaxed Uncorrelated Lognormal) and then Bayes Factor was used to select which fitted the data better. The trees obtained with the best clock model were analyzed with BaTS in order to calculate the Parsimony Score (PS), the Association Index (AI) (for overall association) and the Monophyletic Clade (MC) (for each state) statistics. The expected value of the indexes under the non-association hypothesis was estimated by 100 randomized sets.

## Results

### Infected population

A total of 75 sera from HCV-infected patients were analyzed. Forty-nine NS5B sequences were obtained for patients from CdE and 26 for patients from OLC. The number of infected patients from CdE increased with age, with a mean age ± S.E.M. of 66.15±1.52 years. Only a few cases (4 out of 49) were detected in patients under 50 years old, whereas 12 infected patients were detected in the 50–60-year old group and other 12 in the 60–70-year old group. Finally, 21 infected patients were either 70 years old or older. In contrast, 18 out of the 26 patients from OLC were aged between 40 and 60 years old. In OLC, the mean age of infected patients was 49.77±2.15.

The difference between means (16.38±2.64 years) was statistically significant, with a **p**<0.0001 (Student *t*-test). Both populations passed the D'Agostino & Pearson Normality Test and their variances were not significantly different (Table 2).

**Table 2:** Number and Location of Sequences.

	NS5B sequences	E2 sequences	Mean Age of Patients (SEM)*
Cruz del Eje	49	22	66.15 (1.52)
Other Locations of Córdoba	26	15	49.38 (2.63)

*Mean Age calculated with patient's age at 2004. **SEM**: Standard Error of the Mean.

### Genbank Accession Numbers

The sequences obtained in this study were deposited in the Genbank under the following accession numbers: JF511020 to JF511061 for the E1/E2 region and JF511062 to JF511136 for the NS5B region.

### Phylogenetic Analyses

Phylogenetic analyses were performed using sequences from the NS5B and E2 regions with the Maximum Likelihood, Neighbor Joining and Parsimony methods.

**Figure 1:**
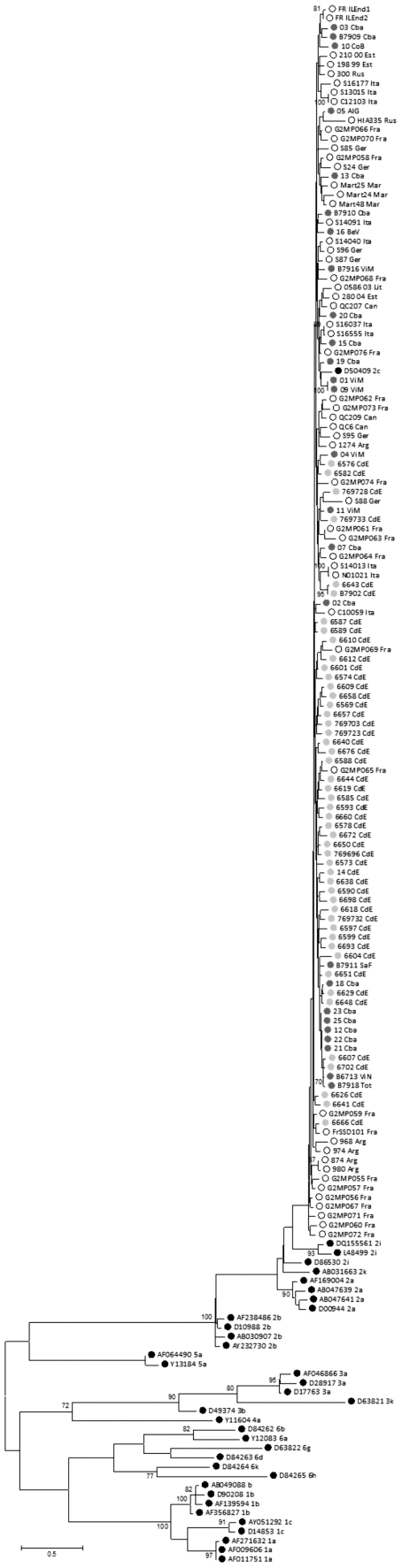
Maximum likelihood tree for the NS5B region constructed using TVM+Gamma+I as model of nucleotide substitution with parameters suggested by ModelTest 3.7 (PhyML software). **Black bullets**: Sequences from the Genotype Reference dataset; **Hollow bullets**: Sequences from the HCV-2c dataset. **Light gray bullets**: Sequences from the CdE data set. **Dark gray bullets**: Sequences from the OLC data set, **Numbers above branches**: bootstrap values over 100 pseudoreplica. Scale bar represents substitution per site.

The analyses were carried out using NSB5 sequences in a combined data set which contained the data sets from CdE (49 sequences)+OLC (26 sequences)+HCV-2c (61 sequences)+Genotype Reference (36 sequences). The Maximum Likelihood phylogenetic reconstruction of these sequences (Figure 1) showed well-supported groups for each genotype and for subtype 2a (bootstrap value of 87). Subtypes 2b and 2i also formed clusters, although poorly supported. The CdE, OLC and HCV-2c sequences clustered with the HCV-2c reference sequence, forming a monophyletic group. When analyzed by the Neighbor Joining and Parsimony methods, the tree topology obtained for the same data set was similar to that obtained by Maximum Likelihood. In the case of the tree obtained by Neighbor Joining, the subtypes had higher bootstrap values and the HCV-2c cluster was supported with a value of 86 (Figure S1). The parsimony analysis yielded 56 most parsimonious trees with a total length of 2407 steps. These trees were used to construct a strict consensus tree which also showed clear subtype clustering and grouped all the sequences from the Province of Córdoba with those already genotyped as HCV-2c in a monophyletic (but collapsed) cluster (Figure S2).

The E2 region was analyzed with a combined data set containing the data sets from CdE (27 sequences)+OLC (15 sequences)+Genotype Reference (34 sequences), yielding results similar to those obtained for the NS5B region, although higher bootstrap values in the E2 phylogeny and better support of the subtype clusters were observed (Figure S3). Geographical association could not be assessed for this region since the only HCV-2c E2 sequence spanning the region analyzed outside the Province of Córdoba belonged to the Genotype Reference data set.

### Phylogeography Analysis

The relaxed uncorrelated lognormal clock model was selected by analyzing the Bayes factor of BSPs. The overall association between geographic traits and phylogeny was assessed by AI and PS (Table 3). Both indexes showed statistically significant (**p**<0.001) differences with the null distribution (i.e., no association). Interestingly, the state-specific index MC showed a significant (p<0.05) association for Italy, Argentina (places other than Córdoba Province), France, Martinique, Other Locations of Córdoba Province and Cruz del Eje.

**Table 3:** Summary of association index for phylogeographic analysis.

Statistics	observed mean	lower 95% CI	upper 95% CI	null mean	lower 95% CI	upper 95% CI	Significance level
AI	6.065	4.999	7.129	12.734	11.934	13.472	**<0.001**
PS	46.047	42.000	50.000	74.969	72.451	77.384	**<0.001**
MC (CdE)	10.658	6.000	18.000	3.063	2.552	4.028	**0.010**
MC (OLC)	4.811	3.000	5.000	2.072	1.590	2.867	**0.010**
MC (Martinique)	2.135	1.000	3.000	1.019	1.000	1.067	**0.010**
MC (France)	4.002	2.000	7.000	1.934	1.442	2.609	**0.020**
MC (Argentina)	2.227	2.000	4.000	1.051	1.000	1.238	**0.020**
MC (Italy)	2.191	2.000	3.000	1.240	1.028	1.943	**0.040**
MC (Esthonia)	1.218	1.000	2.000	1.011	1.000	1.041	1.000
MC (Germany)	1.181	1.000	2.000	1.091	1.001	1.659	1.000
MC (Russia)	1.030	1.000	1.000	1.006	1.000	1.009	1.000
MC (Canada)	1.001	1.000	1.000	1.008	1.000	1.036	1.000
MC (Lithuania)	1.000	1.000	1.000	1.000	1.000	1.000	1.000

Results of Bayesian Tips Significance Tests (BaTS). Association Index (AI) and Parsimony Score (PS) tests the global association between a trait and tree topology taking into account the level of uncertainty in the phylogenetic reconstruction. The Monophyletic Clade (MC) index allows to test whenever each trait is associated with phylogeny. The observed mean and its associated 95% confidence intervals (Upper and Lower CI) were obtained by analyzing trees sampled during the MCMC (Bayesian phylogenetic reconstruction). The null mean and its associated confidence intervals were obtained after randomly distributing the traits in the phylogeny (100 replica). Significance level is the p value for the statistical hypothesis test for equality between the observed index and that expected under no-association.

### Coalescent Analyses

The estimation of t_MRCA_ was carried out by coalescent analysis of the following NS5B data sets and combinations: 1) CdE; 2) OLC; 3) Fr; 4) CdE+OLC; 5) CdE+OLC+Fr.

For all data sets, the relaxed clock model performed better than the strict one by analyzing the Bayes factor of BSPs. The t_MRCA_ (expressed in years before last sampling date) for the analyses were between 107 y (OLC data set) and 138 y (CdE+OLC+Fr combined data set) (Table 4).

**Table 4:** Summary for t_MCRA_ estimations using the NS5B region.

Dataset	Mean t_MRCA_ (SEM)	Median t_MRCA_	HPD-95%(lower)	HPD-95%(upper)
CdE	115.51 (0.44)	111.12	68.85	168.54
OLC	111.71 (0.52)	107.77	69.99	162.62
Fr	102.12 (0.49)	99.37	66.21	143.79
CdE+OLC	141.15 (0.55)	135.87	87.62	207.06
CdE+OLC+Fr	143.46 (0.43)	138.46	88.71	210.06

t_MRCA_ estimations for NS5B data sets. Results correspond to Bayesian Skyline Plots run under the GTR+Γ+I model of nucleotide substitution using a relaxed clock model (uncorrelated lognormal) and setting a rate of nucleotide substitution of 5×10^−4^ substitutions per site per year as prior. **CdE**: Cruz del Eje sequences; **OLC**: Other Locations of Córdoba sequences; **Fr**: Genbank HCV-2c NS5B sequences from France; **CdE+OLC**: combined data set containing CdE and OLC sequences; **CdE+OLC+Fr**: combined data set containing CdE and CdE and Fr sequences; **t_MRCA_**: time of most recent common ancestor (years before present); **HPD-95%**: High Posterior Probability Density 95% (years before present); **SEM**: Standard Error of the Mean.

**Figure 2:**
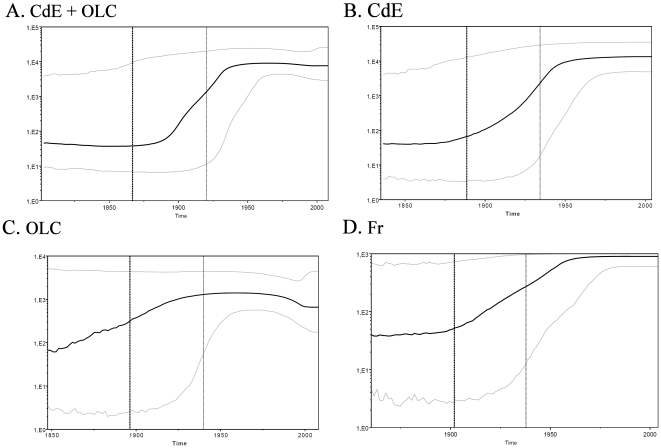
Bayesian Skyline Plots for Demographic Reconstruction using NS5B sequences. **X axis**: Date in Years A. C.; **Y axis**: Estimated effective number of infections; **Bold Dashed Line**: Median time of most recent common ancestor (t_MRCA_t_MCRA_); **Light Dashed Line**: Upper HPD95% of t_MRCA_. **Bold Line**: Mean Effective Number of viral population. **Blue Lines**: Upper and Lower HPD95% of Effective Number of viral population. Analyzed data sets: **A. CdE+OLC**: Samples from Cruz del Eje and Other locations of Córdoba Province; **B. CdE**: Samples from Cruz del Eje; **C. OLC**: Samples from other locations of Córdoba Province; **D. FR**: Samples from South Western France (Cantaloube *et al.*, 2008).

In addition, the skyline plot profiles for all the data sets analyzed, except for the OLC one, were in a steady non-expanding “lag” phase up to about 1880, then grew exponentially up to the 1940s, and finally were again in a lag phase after the 1960s (Figure 2 A, B and D). The OLC BSP showed little expansion of the population size since the t_MRCA_ up to the 1940s, then the population remained constant until the 1980s and finally it seems to be declining until the present time (Figure 2 C).

The substitution rate was also estimated by coalescent analysis of the following E2 data sets and combinations: 1) CdE; 2) OLC; 3) CdE+OLC.

For each data set, the t_MRCA_ was set as a prior with gamma distribution, where the shape and the scale parameters were set according to the distribution of the previously estimated t_MRCA_ of the corresponding NS5B data set. Since there are samples with sequences only for the NS5B region, the t_MRCA_ was re-estimated using reduced data sets containing only NS5B sequences that had its E2 counterparts. It is not surprising that t_MRCA_ estimation with the full and the reduced data sets was almost the same (Data S1 (Raw_TMRCA_estimation.xls)).The t_MRCA_ gamma prior set for each data set, described by its median (5% and 95% percentiles) in years before present were: 106.9 (67.0 to 152.5) for the CdE data set, 108.9 (71.2 to 158.0) for the OLC data set and 131.1 (86.4 to 189.2) for the CdE+OLC data set. The estimated substitution rates for the E2 region (expressed in s/s/y, high posterior probability density 95% in parentheses) were: 1.14×10^−3^ (0.63×10^−3^ to 1.83×10^−3^) for the CdE data set, 1.77×10^−3^ (0.96×10^−3^ to 2.96×10^−3^) for the OLC data set and 1.87×10^−3^ (1.02×10^−3^ to 3.11×10^−3^) for the CdE+OLC data set (Table 5). The skyline profiles of the CdE, OLC and CdE+OLC data sets were compatible with those obtained by the NS5B region. They showed a lag phase until 1900 followed by exponential growth until 1950–1960 and then again a constant population size until the present (Figure S4).

**Table 5:** Summary for E2 substitution rate estimations.

Dataset	Mean Substitution Rate	Standard Error of mean	Median Substitution Rate	HPD-95% (lower)	HPD-95% (upper)
	(×10^−3^ s/s/y)	(**×**10^−5^)	(**×**10^−3^ s/s/y)	(**×**10^−3^ s/s/y)	(**×**10^−3^ s/s/y)
OLC	1.86	1.16	1.77	0.96	2.96
CdE	1.19	0.63	1.14	0.64	1.83
CdE+OLC	1.97	1.61	1.87	1.02	3.11

Substitutions rate for E2 data sets. Results correspond to Bayesian Skyline Plots run under appropriate model of nucleotide substitutions: GTR+Γ+I for CdE+OLC and CdE data sets and HKY+Γ+I for OLC data set. Relaxed clock model (uncorrelated lognormal) was used. The t_MRCA_ for each data set was set as prior with gamma distribution (Shape; Scale): 18.03; 7.41 for CdE+OLC data set; 17.45; 6.36 for OLC data set; 16.42; 6.46 for CdE data set. **CdE**, Cruz del Eje E2 sequences, **OLC**, Other Locations of Córdoba E2 sequences. **CdE+OLC**, sequences from CdE and OLC data sets. **s/s/y**: substitutions per site per year; **HPD-95%**, High Posterior Probability Density 95%.

## Discussion

It has been proposed that HCV Genotype 2 may have originated in Western Africa [5]. The role of human migration in the early and recent spread of some HCV subtypes, such as HCV-2c, has been evidenced in recent works in European populations [6]. Although most HCV-2c sequences deposited in Genbank were obtained from samples from Europe, mainly France and Italy [8], [15], this may not reflect t a possible European origin of the sequences, but rather that these countries have carried out more detailed epidemiological studies. Interestingly, certain subtypes like 2a, 2b and 2c have a worldwide distribution, possibly associated with a relatively recent dissemination through blood transfusions, surgical practices and intravenous drug abuse. However, in some countries, such as Italy, HCV-2c is an epidemiological important subtype, being the second most prevalent (after HCV-1b) with the particularity that it forms an increasing north-to-south gradient of prevalence and it is associated with elder patients [9]. In Cruz del Eje city, HCV-2c accounts for 90% of HCV infections, whereas in other cities and towns of the Province of Córdoba it accounts for 50% [11], [12]. The fact that 21 out of the 49 sequenced samples from Cruz del Eje belong to patients older than 70 years, as that observed in Italy, suggests that the spread of HCV in the population was a past event. A different situation was observed in other towns and cities of the Province of Córdoba (including Córdoba city), where most of the infected patients were between 40 and 50 years old (in 2004), which suggests that the dissemination occurred more recently.

In order to trace back the origin and dissemination of HCV-2c in the Province of Córdoba, phylogenetic analysis was performed using the Maximum Likelihood, Parsimony and Neighbor Joining methods. In all cases, the sequences from Córdoba consistently formed a monophyletic group with the sequences from all the countries where HCV-2c has been sequenced. A similar analysis was carried out with the E2 region and, again, the samples from Córdoba also grouped with the HCV-2c reference genomes.

The demographic history was estimated by coalescent analysis of NS5B sequences. The Bayesian Skyline Plot was set as a demographic model because it allows the reconstruction of the population dynamics without imposing any parametric model [25]. Since the samples from this study represent less than 10 years of history (i.e. the samples are almost contemporaneous), a previously calculated substitution rate of 5×10^−4^ s/s/y [21] was used. Unfortunately, the use of an external rate to calibrate the relaxed molecular clock has some drawbacks. One of these drawbacks is that it has to be set as a global rate including all CdE and OLC samples even when these populations have a different patient's age and may have different intra-host evolution time, leading to different substitution rates. This drawback is, to some extent, avoided by using a relaxed molecular clock, which allows the rate to vary among lineages. Another drawback is that it has to be set as a prior distribution (i.e. normal distribution) with a high standard deviation which leads to broad estimation errors (HPD95% limits). Thus, the results have to be taken with care. Under these priors, the data sets analyzed yielded a time for the most recent common ancestor (t_MRCA_) ranging from 107.8 years for OLC to 138.5 years for CdE+OLC+Fr, but since all HPD95% intervals included the medians of the other data sets, there were no significant differences between the estimated t_MRCA_s. It is worth noting that when the analyses are carried out using recent re-calculated substitution rates (for exactly the same genomic fragment) between 2.5×10^−3^ and 4×10^−3^ s/s/y [27], [28], the resulting t_MRCA_s are about 30 to 40 years. This estimation appears too recent to fit with the epidemiological information such as the fact that 21 out of 49 infected patients are at least 70 years old.

The skyline plot profiles using 5×10^−4^ s/s/y as substitution rate showed an exponential growth of the effective number of infections for the CdE and Fr data sets for a period of about 60 years (from 1880–1890 to 1940–1950), after which it remained constant. It is worth noting that the upper (HPD95%) limit for population size in the four data sets remained constantly high, making these BSPs compatible with a constant size population dynamics. However, the changes in the viral population depicted in the NS5B BSP profiles were further supported when the E1/E2 region was analyzed. In this case, the profile obtained was very similar to the corresponding NS5B data set but with narrower HPD95% intervals. Moreover, this hypothesis is supported by the fact that the Argentinean population experienced an important growth (due to immigration) in the period of the exponential growth shown by the skyline. Such a change in the host density is usually associated with the emergence of viral infections [24].

When the BSP analysis was carried out using substitution rates of 2.5×10^−3^ or 4×10^−3^ s/s/y, a similar demographic profile was obtained, although with an exponential period of around 10 years between 1985 and 1995, which again does not fit with the epidemiological information. The results of both t_MRCA_ and BSP analyses highlight the influence of prior selection in the coalescent analysis and encourage the research aimed to obtain a more accurate estimation of the substitution rate.

The global dispersal of HCV and that of several other pathogens show a similar demographic pattern, which may be the consequence of a process associated with blood transfusions, intravenous drug abuse and invasive medical and surgical procedures that have been common during the last 80 years [4].

It has been reported that global dispersal of HCV-1b and HCV-1a has occurred by these means. Moreover, in the case of HCV-1b, the spread of the virus has occurred in a two-step process, first in developed countries, and then in developing countries [26]. In Argentina, which is a developing country, the previously estimated t_MRCA_ for HCV-1b in the cosmopolitan city of Buenos Aires (∼50 years) [28] and in the small town of Wheelwright (∼55 years) [27] were more recent than the t_MRCA_ estimated for HCV-2c.

The phylogeographic analysis showed that samples from CdE, OLC, Italy, France, Argentina and Martinique grouped non-randomly according to their geographical origin, which suggests that the emergence and diversification of these sequences is probably a recent event from ancestral sequences located in each geographical region. A more representative sampling would be required to make further speculations about the global path of HCV-2c transmission.

The history of Argentina shows immigration from regions where HCV-2c is an epidemiological important subtype: Africa and Europe. Although during the 16th–18th centuries Argentina received immigrants from Africa as slaves, the present ethnic composition of the population was strongly affected by the European immigration process (mainly from Italy) occurred during the first half of the 20th century (particularly between 1880 and 1920). The settlement of the Italian immigrants in the central region of Argentina, where the Province of Córdoba is located, was rural. This can account for the current distribution of HCV-2c with higher prevalence and patient's age in the “rural” city of Cruz del Eje, compared with the “cosmopolitan” city of Córdoba.

In summary, the origin of HCV-2c circulating in Argentina may be related to the European immigration. The lack of HCV-2 subtypes other than 2c and the presence of HCV-1b, together with the similar distribution in the age of infected patients and the migration history of Argentina, suggest that Italy might be the source of the HCV-2c circulating in Córdoba. Finally, these results suggest that HCV-2c simultaneously suffered an emerging process, perhaps associated with those medical care factors mentioned above (i.e. blood transfusions, injectable treatments, etc.).

The study of the genetic diversity of HCV circulating in different countries and regions not only provides useful information for the local epidemiology and diagnosis but also serves as another piece on the puzzle of HCV evolution.

## Supporting Information

Figure S1 **Neighbor Joining Tree for the NS5B region.** The nucleotide substitutions process was modeled with Maximum Composite Likelihood (TrN93) model (MEGA4 software). **Black bullets**: Sequences from the Genotype Reference dataset; **Hollow bullets**: Sequences from the HCV-2c dataset. **Light gray bullets**: Sequences from the CdE data set. **Dark gray bullets**: Sequences from the OLC data set, **Numbers above branches**: bootstrap values over 1000 pseudoreplica. Scale bar represents substitution per site.
(TIF)

Figure S2 **Strict Consensus tree constructed using the 56 most parsimonious trees (2407 steps) found using the New Technology Search (TNT software) for the NS5B region. Black bullets**: Sequences from the Genotype Reference dataset; **Hollow bullets**: Sequences from the HCV-2c dataset. **Light gray bullets**: Sequences from the CdE dataset. **Dark gray bullets**: Sequences from the OCL dataset, **Numbers above branches**: bootstrap values over 100 pseudoreplica. Scale bar represents substitution per site.
(TIF)

Figure S3 **Maximum likelihood tree for the E2 region constructed using GTR+Γ+I as model of nucleotide substitution with parameters suggested by ModelTest 3.7 (PhyML software). Black bullets**: Sequences from the Genotype Reference dataset; **Light gray bullets**: Sequences from the CdE data set. **Dark gray bullets**: Sequences from the OLC data set, **Numbers above branches**: bootstrap values over 100 pseudoreplica. Scale bar represents substitution per site.
(TIF)

Figure S4 **Bayesian Skyline Plots for Demographic Reconstruction using E1E2 sequences. X axis**: Date in Years A. C.; **Y axis**: Estimated effective number of infections; **Bold Dashed Line**: Median time of most recent common ancestor (t_MRCA_); **Light Dashed Line**: Upper HPD95% of t_MRCA_. **Bold Line**: Mean Effective Number of viral population. **Blue Lines**: Upper and Lower HPD95% of Effective Number of viral population. Analyzed data sets: **A. CdE**: Samples from Cruz del Eje; **B. OLC**: Samples from other locations of Córdoba Province; C. **CdE+OLC**: Samples from Cruz del Eje and Other locations of Córdoba Province.
(TIF)

Data S1 **Raw Results tMRCA Estimations for NS5B region.**
(XLS)
