# Supplementary figures and images for: Phylodynamics of Hepatitis C Virus Subtype 2c in the Province of Córdoba, Argentina
Source: PLoS One. 2011 May 18;6(5):e19471. doi: 10.1371/journal.pone.0019471 (PMC3097208; doi:10.1371/journal.pone.0019471)

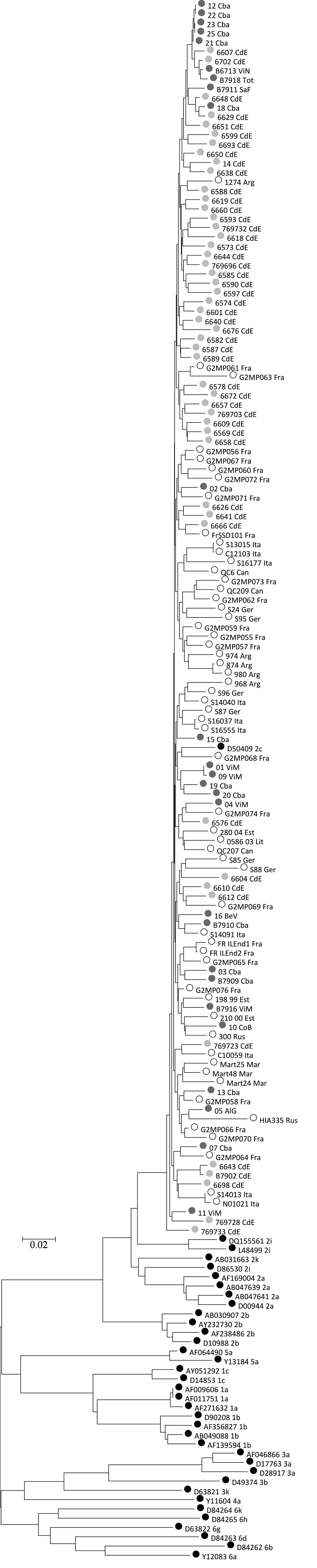

Supplement: Figure S1 — Neighbor Joining Tree for the NS5B region. The nucleotide substitutions process was modeled with Maximum Composite Likelihood (TrN93) model (MEGA4 software). Black bullets: Sequences from the Genotype Reference dataset; Hollow bullets: Sequences from the HCV-2c dataset. Light gray bullets: Sequences from the CdE data set. Dark gray bullets: Sequences from the OLC data set, Numbers above branches: bootstrap values over 1000 pseudoreplica. Scale bar represents substitution per site. (TIF) [file pone.0019471.s001.tif]

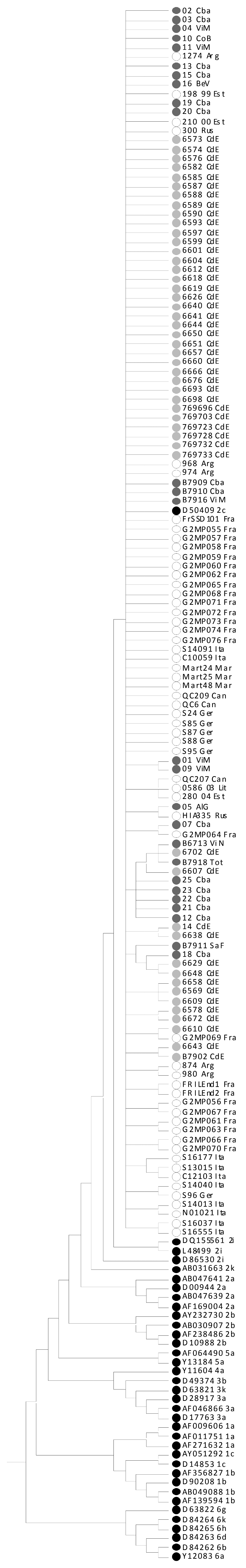

Supplement: Figure S2 — Strict Consensus tree constructed using the 56 most parsimonious trees (2407 steps) found using the New Technology Search (TNT software) for the NS5B region. Black bullets: Sequences from the Genotype Reference dataset; Hollow bullets: Sequences from the HCV-2c dataset. Light gray bullets: Sequences from the CdE dataset. Dark gray bullets: Sequences from the OCL dataset, Numbers above branches: bootstrap values over 100 pseudoreplica. Scale bar represents substitution per site. (TIF) [file pone.0019471.s002.tif]

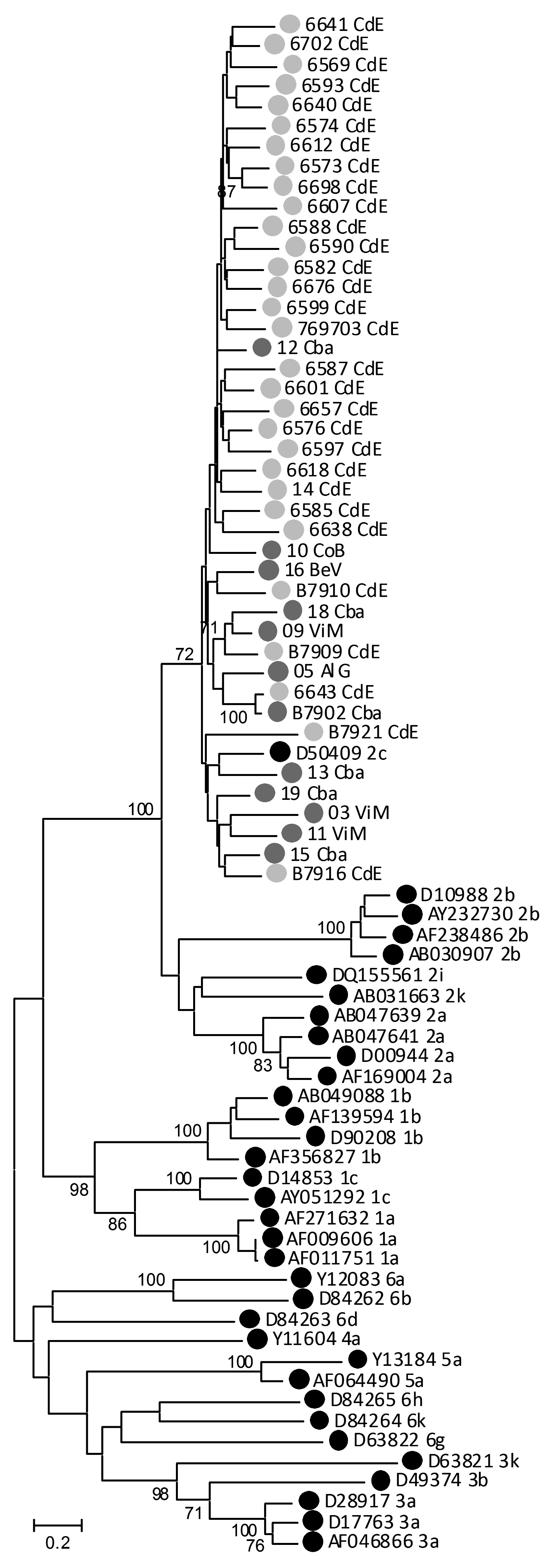

Supplement: Figure S3 — Maximum likelihood tree for the E2 region constructed using GTR+Γ+I as model of nucleotide substitution with parameters suggested by ModelTest 3.7 (PhyML software). Black bullets: Sequences from the Genotype Reference dataset; Light gray bullets: Sequences from the CdE data set. Dark gray bullets: Sequences from the OLC data set, Numbers above branches: bootstrap values over 100 pseudoreplica. Scale bar represents substitution per site. (TIF) [file pone.0019471.s003.tif]

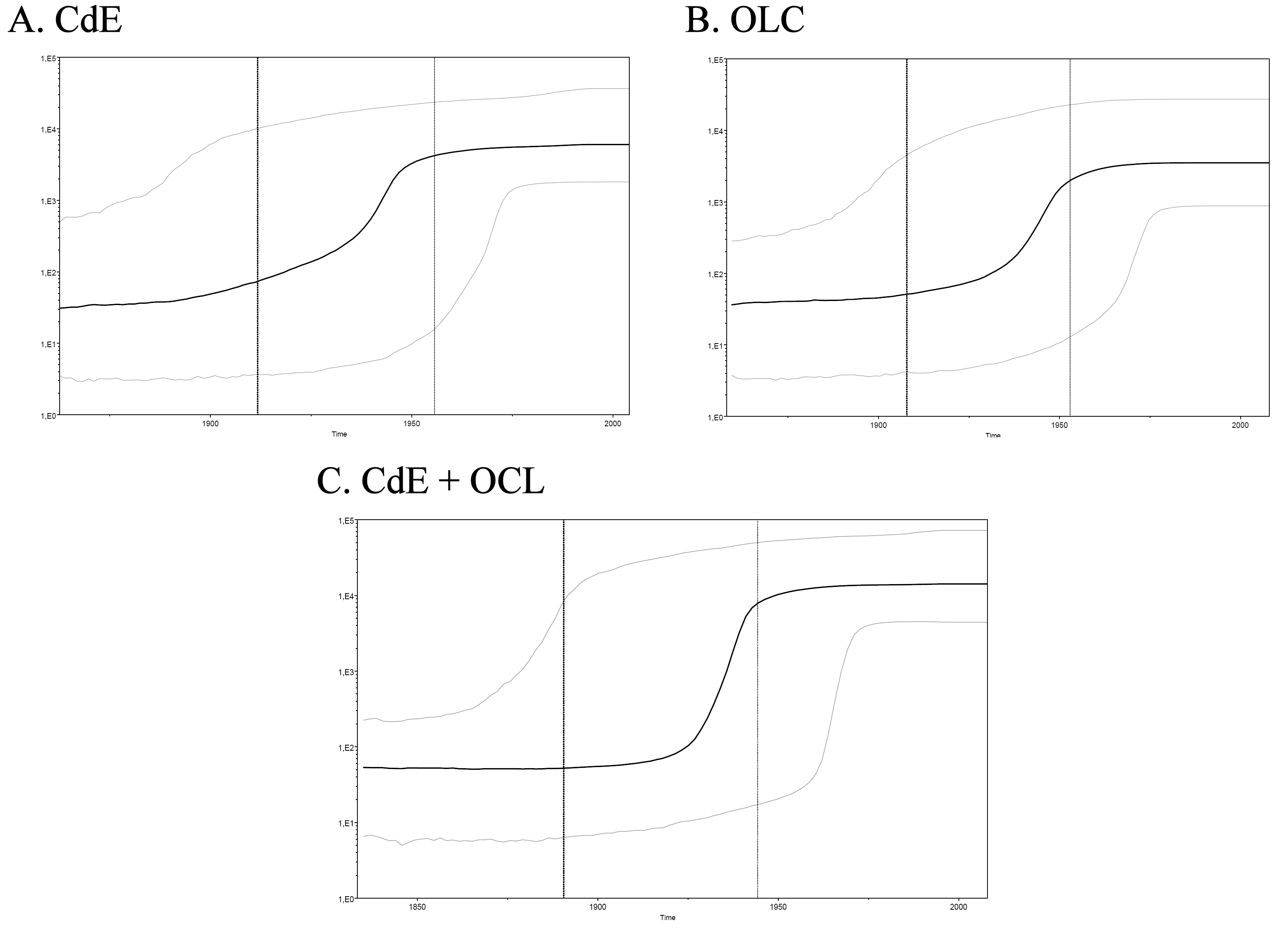

Supplement: Figure S4 — Bayesian Skyline Plots for Demographic Reconstruction using E1E2 sequences. X axis: Date in Years A. C.; Y axis: Estimated effective number of infections; Bold Dashed Line: Median time of most recent common ancestor (tMRCA); Light Dashed Line: Upper HPD95% of tMRCA. Bold Line: Mean Effective Number of viral population. Blue Lines: Upper and Lower HPD95% of Effective Number of viral population. Analyzed data sets: A. CdE: Samples from Cruz del Eje; B. OLC: Samples from other locations of Córdoba Province; C. CdE+OLC: Samples from Cruz del Eje and Other locations of Córdoba Province. (TIF) [file pone.0019471.s004.tif]
